# Successful Management of Severe Hepatic Acute Graft‐Versus‐Host Disease After Allogeneic Hematopoietic Stem Cell Transplantation in a Child With β‐Thalassemia Major: Clinical Lessons From Early Therapeutic Escalation

**DOI:** 10.1002/ccr3.73623

**Published:** 2026-09-25

**Authors:** Hind Alhiraki, Amal Ali Fares, Maged Kheder

**Affiliations:** ^1^ Department of Pediatrics Children's University Hospital, Faculty of Medicine, Damascus University Damascus Syria; ^2^ Pediatric Hematopoietic Stem Cell Transplantation Center, Head of National Stem Cell Center (HAYAT) Children's University Hospital, Faculty of Medicine, Damascus University Damascus Syria

**Keywords:** β‐thalassemia major, acute graft‐versus‐host disease, hepatic‐predominant GVHD, immunosuppressive therapy, pediatric hematopoietic stem cell transplantation, ruxolitinib

## Abstract

Severe hepatic acute graft‐versus‐host disease can occur early after allogeneic hematopoietic stem cell transplantation without skin involvement, presenting with rapidly progressive cholestatic liver dysfunction. Early recognition and prompt escalation to multimodal immunosuppressive therapy achieve complete biochemical remission and durable hematopoietic recovery, highlighting the crucial role of early, multidisciplinary pediatric management.

## 1 Introduction

β‐Thalassemia major is one of the most common inherited hemoglobin disorders worldwide and represents a major public health problem in many regions, particularly in the Mediterranean, Middle East, and Southeast Asia. The disease is characterized by defective β‐globin chain synthesis, leading to ineffective erythropoiesis, chronic anemia, and the need for lifelong regular blood transfusions to maintain adequate hemoglobin levels and normal growth in affected children [1].

Chronic transfusion therapy combined with iron chelation has significantly improved survival and quality of life in patients with transfusion‐dependent thalassemia. However, long‐term complications, particularly iron overload and its cardiac, hepatic, and endocrine consequences, remain major challenges in disease management. Allogeneic hematopoietic stem cell transplantation (HSCT) remains the only established curative treatment for transfusion‐dependent β‐thalassemia [2].

Despite its curative potential, HSCT is associated with several serious complications. Among these, graft‐versus‐host disease (GVHD) remains an important cause of morbidity and mortality following transplantation. Acute GVHD results from donor‐derived immunocompetent T‐cell recognition of recipient tissues, leading to inflammatory injury that primarily involves the skin, gastrointestinal tract, and liver. The development and severity of GVHD are influenced by multiple factors, including donor‐recipient characteristics, conditioning regimen, graft source, and GVHD prophylaxis [3, 4, 5, 6].

Hepatic involvement in acute GVHD commonly occurs in conjunction with gastrointestinal and/or cutaneous manifestations. However, hepatic‐predominant GVHD without prominent skin involvement may present a diagnostic challenge, particularly during the early post‐transplant period. Significant hepatic dysfunction after HSCT also requires careful consideration of alternative diagnoses, including sinusoidal obstruction syndrome/veno‐occlusive disease (SOS/VOD) and transplantation‐associated thrombotic microangiopathy (TA‐TMA) [7, 8, 9].

Severe acute GVHD represents a major therapeutic challenge. Systemic corticosteroids remain the standard first‐line treatment, whereas patients with steroid‐refractory or inadequately responsive disease may require additional immunosuppressive or targeted therapies [5, 6]. Ruxolitinib, a selective Janus kinase (JAK1/JAK2) inhibitor, has emerged as an important therapeutic option for steroid‐refractory acute GVHD and has demonstrated clinically meaningful activity in both adult and pediatric populations [10, 11].

Herein, we report the case of a pediatric patient with transfusion‐dependent β‐thalassemia major who developed severe acute GVHD with predominant hepatic and gastrointestinal involvement following allogeneic HSCT from a fully matched sibling donor. Notably, the patient developed severe cholestatic hepatic involvement without cutaneous manifestations. The condition was managed with intensive multimodal immunosuppressive therapy, including ruxolitinib and rituximab, resulting in complete clinical and biochemical recovery and sustained remission during 1 year of follow‐up.

## 2 Case Presentation

A 4.5‐year‐old boy with transfusion‐dependent β‐thalassemia major was diagnosed at the age of 3 months based on hemoglobin electrophoresis findings (HbA1 15%, HbA2 3%, HbF 82%). He was maintained on regular monthly blood transfusions combined with iron chelation therapy. At the age of 4 years, the patient was evaluated for allogeneic hematopoietic stem cell transplantation (HSCT) at Hayat Stem Cell Transplantation Center, Syria. HLA typing confirmed a fully matched sibling donor. Pre‐transplant evaluation included viral, cardiac, pulmonary, dental, ENT, and psychological assessments. Whole‐body computed tomography was unremarkable except for increased hepatic density. Liver biopsy demonstrated Grade I parenchymal iron overload without portal fibrosis or hemosiderin granules. According to the Pesaro classification, the patient was categorized as Class I. Hemoglobin electrophoresis results for the patient and family members are summarized in Table 1.

**TABLE 1:** Hemoglobin electrophoresis results of the patient and family members before hematopoietic stem cell transplantation (HSCT).

	A1	A2	F
The patient	15	3	82
His mother	94.7	5.3	
His father	95.2	4.8	
His sister	97.9	2.1	

The conditioning regimen followed the EBMT protocol and included busulfan (4.4 mg/kg/day for 4 days), cyclophosphamide (50 mg/kg/day for 4 days), and anti‐lymphocyte globulin (Grafalon, 15 mg/kg/day for 2 days). Peripheral blood stem cells were collected from the donor, with a total graft volume of 140 mL containing 11 × 10^6^ CD34+ cells/kg and 580 × 10^6^ CD3+ cells/kg. GVHD prophylaxis consisted of methotrexate (10 mg/m^2^ on day +1 and 6 mg/m^2^ on days +3 and +6, with leucovorin rescue) in combination with cyclosporine A (initially 1.5 mg/kg/day, increased to 3 mg/kg/day). Supportive care included irradiated leukocyte‐filtered blood products, acyclovir, and trimethoprim‐sulfamethoxazole prophylaxis. Neutrophil engraftment was achieved on day +15 and platelet engraftment on day +13. Donor chimerism analysis on day +30 demonstrated 99.2% donor cells. On day +23 post‐transplant, the patient developed fatigue, progressive jaundice, and hepatomegaly extending 4 cm below the right costal margin, without cutaneous manifestations. He was diagnosed with Grade III acute hepatic‐predominant GVHD with concurrent Grade II gastrointestinal GVHD. Laboratory investigations at the time of diagnosis revealed WBC 4000/μL, ANC 3200/μL, hemoglobin 11.5 g/dL, platelet count 320,000/μL, ALT 250 U/L, AST 200 U/L, GGT 936 U/L, ALP 425 U/L, albumin 3.7 g/dL, LDH 250 U/L, creatinine 0.2 mg/dL, PT 90%, and PTT 36 s. The laboratory profile demonstrated marked cholestatic liver dysfunction, with bilirubin levels progressively increasing and peaking at ≥ 20 mg/dL during the acute GVHD episode. Viral investigations, including hepatitis virus screening and cytomegalovirus (CMV) testing, were negative.

The differential diagnosis of early post‐transplant hepatic complications was carefully considered. Transplant‐associated thrombotic microangiopathy (TA‐TMA) was considered less likely in the absence of laboratory and clinical features suggestive of microangiopathic hemolysis or renal involvement. At admission to the transplant unit, the platelet count was 240,000/μL and serum creatinine was 0.6 mg/dL, and both remained within the normal range during the subsequent clinical course. LDH was normal, peripheral blood smear showed no schistocytes, blood pressure remained normal throughout the episode, and urinalysis showed no proteinuria. Sinusoidal obstruction syndrome/veno‐occlusive disease (SOS/VOD) was also considered. However, the patient had no ascites or abdominal distension and showed no significant weight gain; his weight remained stable between 15.3 and 15.5 kg from transplantation through clinical recovery. Abdominal Doppler ultrasonography showed normal hepatic echogenicity, with mild dilatation of the hepatic veins and a mild increase in portal venous pressure. Taken together with the clinical and laboratory findings, the absence of significant weight gain, ascites, abdominal distension, thrombocytopenia, renal dysfunction, microangiopathic changes, hypertension, or proteinuria made TA‐TMA and SOS/VOD less likely. The marked cholestatic dysfunction, hepatomegaly, concurrent gastrointestinal GVHD, absence of cutaneous involvement, and subsequent response to GVHD‐directed therapy supported acute hepatic‐predominant GVHD as the most likely diagnosis.

## 3 Treatment

At GVHD onset, the patient weighed 15.3 kg, and all medications were dosed accordingly. Initial treatment consisted of prednisolone (2 mg/kg/day), cyclosporine A (5 mg/kg/day), azathioprine (3 mg/kg/day), ursodeoxycholic acid (40 mg/kg/day), silymarin (15 mg/kg/day), cholestyramine (3 g/day), and intravenous acetylcysteine (150 mg/kg/day in divided doses). Despite this initial multimodal treatment, the patient showed limited clinical and biochemical response, with persistent severe jaundice and cholestatic liver dysfunction. Given the severity and persistence of the hepatic involvement, immunosuppressive therapy was subsequently intensified by adding mycophenolate mofetil (50 mg/kg/day), ruxolitinib (5 mg/day), and rituximab (375 mg/m^2^ intravenously once weekly).

Following treatment escalation, the patient demonstrated progressive clinical and laboratory improvement. Within 3 weeks, jaundice and hepatomegaly markedly regressed, liver function tests improved substantially, and gastrointestinal symptoms resolved. Subsequent follow‐up showed complete normalization of liver biochemical parameters and sustained remission of GVHD manifestations.

## 4 Follow‐Up

The patient was monitored regularly for 1 year following transplantation. Donor chimerism remained stable throughout follow‐up, measuring 100% at 3 months, 99% at 6 months, 99.4% at 9 months, and 99% at 12 months. Hematologic parameters, including WBC count, ANC, hemoglobin level, and platelet count, remained within normal ranges. Liver function tests showed marked improvement, with ALT decreasing from 250 to 35 U/L, AST from 200 to 32 U/L, GGT from 936 to 45 U/L, and bilirubin levels returning to normal. Albumin levels remained stable at approximately 3.7 g/dL, while renal function remained normal throughout follow‐up. No subsequent clinical or laboratory evidence suggestive of thrombotic microangiopathy was observed.

Hemoglobin electrophoresis performed at 9 and 12 months after transplantation demonstrated sustained donor‐type hemoglobin production, with HbA1 of 97% and HbA2 of 2.9%.

Clinically, the patient demonstrated complete resolution of jaundice, hepatomegaly, and gastrointestinal symptoms. He remained transfusion‐independent throughout follow‐up. Immunosuppressive therapy was gradually tapered and eventually discontinued. At the most recent follow‐up, the patient remained healthy and clinically stable and had resumed normal daily activities.

## 5 Discussion

Allogeneic hematopoietic stem cell transplantation (HSCT) remains the only established curative treatment for transfusion‐dependent β‐thalassemia major; however, graft‐versus‐host disease (GVHD) remains one of the most serious complications following transplantation [2]. Acute GVHD most commonly involves the skin, gastrointestinal tract, and liver. Although hepatic involvement may occur as part of multisystem GVHD, hepatic‐predominant disease without associated cutaneous manifestations is less commonly described and may complicate early clinical recognition [3, 7]. Severe acute GVHD represents a major therapeutic challenge, and systemic corticosteroids remain the standard first‐line treatment, while steroid‐refractory or inadequately responsive disease may require additional therapeutic strategies [5, 6].

In the present case, the development of severe cholestatic liver dysfunction with marked hyperbilirubinemia and hepatomegaly on day +23, in the absence of skin manifestations, raised an important diagnostic challenge in the early post‐transplant period. Hepatic GVHD can be difficult to distinguish from other causes of hepatic dysfunction after HSCT, including drug‐induced liver injury, infection, and SOS/VOD [7]. SOS/VOD was considered in this patient because of the early post‐transplant timing and hepatic involvement. However, the patient had no ascites or abdominal distension and no significant weight gain; his weight remained stable between 15.3 and 15.5 kg. Pediatric EBMT criteria emphasize the importance of features such as unexplained weight gain, fluid retention, hepatomegaly, and thrombocytopenia in the clinical assessment of SOS/VOD [8]. In our patient, the absence of significant weight gain, ascites, abdominal distension, or thrombocytopenia made SOS/VOD less likely. Doppler ultrasonography demonstrated normal hepatic echogenicity, with mild hepatic vein dilatation and a mild increase in portal venous pressure; these findings were interpreted in the context of the overall clinical picture rather than as diagnostic evidence for or against SOS/VOD.

TA‐TMA was also considered in the differential diagnosis. The contemporary international consensus definition emphasizes a combination of laboratory and clinical features, including anemia, thrombocytopenia, elevated lactate dehydrogenase (LDH), schistocytes, hypertension, elevated soluble C5b‐9, and proteinuria [9]. In this patient, platelet counts remained within the normal range, LDH was normal, peripheral blood smear showed no schistocytes, blood pressure remained normal, and urinalysis showed no proteinuria. Renal function also remained normal, with a serum creatinine of 0.6 mg/dL at admission to the transplant unit and subsequent values remaining normal. Taken together, the absence of the characteristic clinical and laboratory features of TA‐TMA made this diagnosis less likely.

The diagnosis of hepatic‐predominant acute GVHD was therefore supported by the temporal relationship to transplantation, the marked cholestatic liver dysfunction, hepatomegaly, concurrent gastrointestinal GVHD, absence of cutaneous involvement, exclusion of viral hepatitis and CMV infection, and subsequent response to GVHD‐directed therapy. Nevertheless, the diagnosis should be interpreted as a clinically supported diagnosis rather than a histologically confirmed one. A liver biopsy was not performed during the acute episode because treatment was initiated promptly based on the clinical and laboratory findings. Hepatic GVHD may overlap clinically and histologically with other post‐transplant hepatic disorders, making correlation of clinical, laboratory, and histopathologic findings important when biopsy is feasible and clinically indicated [7].

Early recognition and prompt therapeutic escalation were central to the favorable outcome in this case. Initial treatment consisted of systemic corticosteroid‐based immunosuppression with cyclosporine and additional supportive and hepatoprotective measures.

Because the clinical and biochemical response remained limited, treatment was intensified with mycophenolate mofetil, ruxolitinib, and rituximab. Ruxolitinib has emerged as an important therapeutic option for steroid‐refractory acute GVHD through inhibition of JAK1/JAK2‐mediated inflammatory signaling [10]. Its efficacy has been demonstrated in adult clinical trials, while pediatric studies have provided additional evidence supporting its use in children with refractory GVHD [11, 12].

The therapeutic response observed in this patient is consistent with the growing pediatric experience with ruxolitinib. In an early retrospective pediatric series, Khandelwal et al. reported an overall response rate of 45% at 4 weeks among evaluable children treated with ruxolitinib for steroid‐refractory acute GVHD [11]. More recently, the prospective pediatric REACH4 study reported an overall response rate of 84.4% at day 28 and a durable overall response rate of 66.7% at day 56 [12]. These differences likely reflect variations in patient populations, disease severity, treatment setting, and response definitions. In our patient, complete clinical and biochemical resolution was achieved following multimodal therapeutic escalation including ruxolitinib. However, because multiple immunosuppressive agents were administered concurrently, the contribution of any individual treatment, including ruxolitinib or rituximab, cannot be determined from this single case.

A major limitation of this report is the absence of histopathologic confirmation of hepatic GVHD. In addition, some diagnostic biomarkers that may support the evaluation of TA‐TMA, particularly soluble C5b‐9, were not available. Similarly, haptoglobin was not measured. Therefore, although the clinical course and available laboratory findings strongly supported hepatic‐predominant acute GVHD, competing diagnoses cannot be considered to have been excluded with absolute certainty. The single‐case nature of the report also limits the generalizability of the observed therapeutic response and does not allow conclusions regarding the efficacy of the individual components of the multimodal treatment regimen.

This case provides several clinically relevant lessons. First, severe hepatic acute GVHD may occur without cutaneous manifestations and should therefore remain in the differential diagnosis of significant cholestatic liver dysfunction after HSCT. Second, progressive hepatic dysfunction in the early post‐transplant period warrants systematic evaluation for competing diagnoses, particularly SOS/VOD and TA‐TMA. Third, careful longitudinal assessment of weight, fluid status, platelet count, LDH, renal function, blood pressure, peripheral smear, and urinary protein can provide important supportive information when distinguishing among these complications. Finally, early recognition and timely therapeutic escalation, combined with close multidisciplinary monitoring, may contribute to favorable outcomes in selected patients with severe acute GVHD, including in resource‐limited settings.

The one‐year follow‐up further supports the durability of the clinical response in this patient. Donor chimerism remained stable at 100%, 99%, 99.4%, and 99% at 3, 6, 9, and 12 months, respectively. Liver function tests normalized, hematologic parameters remained stable, and hemoglobin electrophoresis demonstrated sustained donor‐type hemoglobin production. The patient remained completely transfusion‐independent, and immunosuppressive therapy was successfully tapered and discontinued. These findings indicate sustained graft function and a favorable long‐term clinical outcome in this individual patient.

Although conclusions regarding treatment efficacy cannot be drawn from a single case, this report adds to the limited clinical experience with severe hepatic‐predominant acute GVHD in pediatric HSCT recipients. It emphasizes the importance of early recognition, careful differential diagnosis, prompt therapeutic escalation, and close multidisciplinary follow‐up in managing severe GVHD.

## 6 Conclusion

This case highlights the importance of early recognition and careful differential diagnosis of severe hepatic acute graft‐versus‐host disease following allogeneic hematopoietic stem cell transplantation. Hepatic‐predominant GVHD may occur without cutaneous manifestations and can mimic other early post‐transplant hepatic complications, particularly sinusoidal obstruction syndrome/veno‐occlusive disease and transplantation‐associated thrombotic microangiopathy. In this patient, timely therapeutic escalation and close multidisciplinary monitoring were associated with complete clinical and biochemical recovery, sustained donor chimerism, and long‐term transfusion independence. This case adds to the limited pediatric experience with severe hepatic GVHD and provides practical clinical considerations for its recognition and management, particularly in resource‐limited settings.

## Author Contributions

**Hind Alhiraki:** writing – review and editing. **Amal Ali Fares:** writing – review and editing. **Maged Kheder:** supervision.

## Funding

The authors have nothing to report.

## Ethics Statement

Ethics approval was not required for this case report according to the institutional policy.

## Consent

Written informed consent for publication was obtained from the patient's legal guardian.

## Conflicts of Interest

The authors declare no conflicts of interest.

## Data Availability

All relevant data supporting the findings of this case report are included within the article. Additional information is available from the corresponding author upon reasonable request.
